# Supplementary material for: Development of Pranoprofen Loaded Nanostructured Lipid Carriers to Improve Its Release and Therapeutic Efficacy in Skin Inflammatory Disorders
Source: Nanomaterials (Basel). 2018 Dec 7;8(12):1022. doi: 10.3390/nano8121022 (PMC6316124; doi:10.3390/nano8121022)
Supplement: Supplementary file 1 [file nanomaterials-08-01022-s001.pdf]

Supplementary materials

Table S1. Homogenization assays to select factorial design conditions.

Parameters tested					
**Number of cycles of the homogenizer**			2-3-4		
**Pressure (bar)**			600-650-700-750-800-850		
**Total lipid (%)**			4-5-6-8		
Best Results obtained			Results		
Number of cycles	Pressure (bar)	Total lipid (%)	Z-Ave (nm)	PI	ZP (mV)
3	800	5	< 350	< 0.3	< -10

Table S2. Factors and the corresponding levels in experimental design.

Factors	-1.68	-1	0	+1	+1.68
**cPF (%)**	0.16	0.50	1.00	1.50	1.84
**cSL/L (%)**	43.20	50.00	60.00	70.00	76.80
**cTW (%)**	2.16	2.50	3.00	3.50	3.84

Table S3. Design of experiments of PF loaded Lipid NLCs.

	**Independent variables**						**Dependent variables**			
	cPF (%)		cSL/L(%)		cTW (%)		Z-Ave(nm)±SD	PI ±SD	ZP(mV) ±SD	EE(%)±SD
					**Factorial points**					
**F1**	-1	0.50	-1	50.00	-1	2.50	427.70±6.25	0.375±0.045	-9.66± 0.71	98.32±0.22
**F2**	-1	0.50	1	70.00	-1	2.50	257.90±7.69	0.266±0.097	-10.40±0.36	98.46±0.36
**F3**	1	1.50	-1	50.00	-1	2.50	248.40±6.34	0.229±0.046	-10.70±0.44	99.68±0.17
**F4**	1	1.50	1	70.00	-1	2.50	413.13±5.78	0.441±0.078	-9.03±0.41	99.82±0.26
**F5**	-1	0.50	-1	50.00	1	3.50	218.57±8.30	0.393±0.066	-10.33±0.29	98.68±0.10
**F6**	1	1.50	-1	50.00	1	3.50	294.93±10.58	0.260±0.059	-10.19±0.14	97.93±0.28
**F7**	-1	0.50	1	70.00	1	3.50	452.53±5.76	0.603±0.069	-7.88±0.18	99.40±0.21
**F8**	1	1.50	1	70.00	1	3.50	204.57±19.43	0.188±0.070	-8.35±0.36	99.80±0.33
					**Axial Points**					
**F9**	0	1.00	1.68	76.80	0	3.00	222.30±9.74	0.237±0.085	-9.20±0.48	99.61±0.12
**F10**	-1.68	0.16	0	60.00	0	3.00	392.77±6.89	0.435±0.046	-8.73±0.55	99.63±0.11
**F11**	1.68	1.84	0	60.00	0	3.00	206.20±5.44	0.202±0.069	-11.10±0.20	88.79±0.29
**F12**	0	1.00	-1.68	43.20	0	3.00	258.27±16.80	0.220±0.157	-10.90±0.47	84.05±0.37
**F13**	0	1.00	0	60.00	1.68	3.84	239.63±11.67	0.265±0.067	-9.03±0.78	99.19±0.34
**F14**	0	1.00	0	60.00	-1.68	2.16	313.47±2.26	0.234±0.043	-9.00±0.37	99.31±0.20
					**Center Points**					
**F15**	0	1.00	0	60.00	0	3.00	300.30±14.38	0.343±0.024	-9.30±0.69	99.31±0.30
**F16**	0	1.00	0	60.00	0	3.00	249.83±25.20	0.232±0.064	-8.81±0.57	99.23±0.22

Table S4. Average particle size of optimized formulations determined by laser diffraction (LD).

Formulation	**d(0.1)**	**d(0.5)**	**d(0.9)**
NLCs-F3	0.081	0.208	0.754
NLCs-F3-L	0.079	0.196	0.550
NLCs-F9	0.084	0.216	0.679
NLCs-F9-L	0.084	0.214	0.620

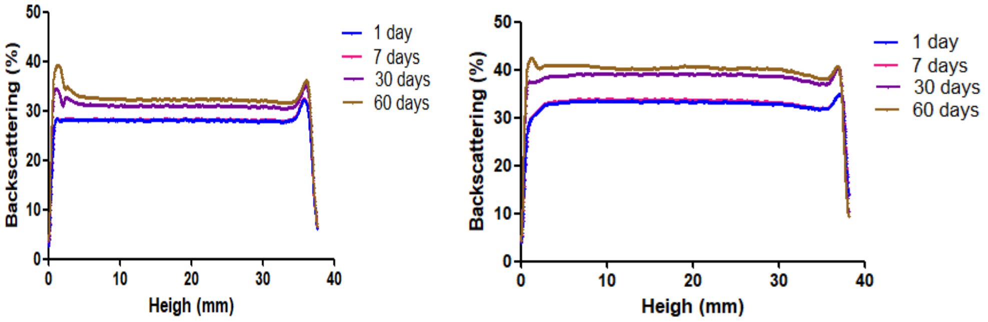

Figure S1. NLCs F-3-L and NLCs F-9-L backscattering profiles obtained by the Turbiscan®Lab stored at 25 °C. The left side of the curve corresponds to the bottom of the vial, whereas the right side corresponds to the sample behavior on the top.

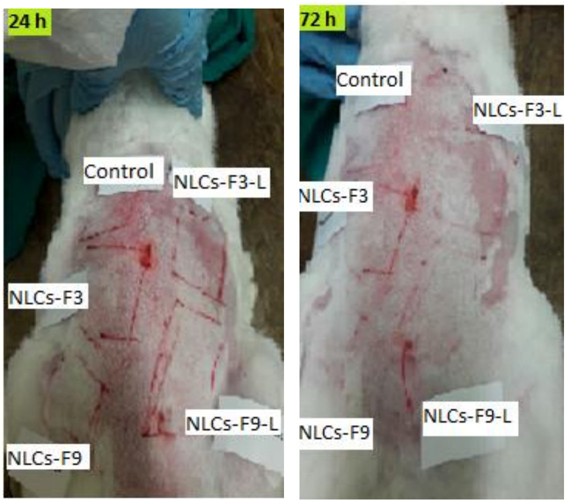

Figure S2. Skin irritation testing. Draize test.
